# Prevalence and disease burden of chronic cough in nine cities of China: an observational study

**DOI:** 10.1186/s12890-024-03017-6

**Published:** 2024-07-04

**Authors:** Lin Chen, Kim S.J. Lao, Fang Yi, Kai Xia, Kefang Lai

**Affiliations:** 1 Global Medical Affairs, MRL, MSD China, Shanghai, China; 2 Global Medical and Scientific Affairs, MSD, Hong Kong, Hong Kong Special Administration Region China; 3 State Key Laboratory of Respiratory Disease, National Clinical Research Center for Respiratory Disease, National Center for Respiratory Medicine, Department of Pulmonary and Critical Care Medicine, Guangzhou Institute of Respiratory Health, The First Affiliated Hospital of Guangzhou Medical University, Guangzhou, China

**Keywords:** Chronic cough, Prevalence, Health-care resource utilization, Medical costs, Claims data

## Abstract

**Background:**

Chronic cough (CC) is common in the general population of China, creating a difficult-to-ignore public health burden. However, there is a lack of research on the nationwide prevalence and disease burden of CC in the Chinese population. We aim to use an insurance claims database to assess the prevalence and the corresponding economic burden owing to CC in China.

**Methods:**

This was a retrospective observational study based on an administrative medical insurance database in 2015, 2016 and 2017, from nine cities in North, South, East, South-West, and North-West regions of China. The study population was Chinese adults (≥ 18 years old) who had been identified as CC patients. Descriptive data analyses were used in statistical analysis.

**Results:**

A total of 44,472, 55,565, and 56,439 patients with mean ages of 53.2 (16.3) years were identified as patients with CC in 2015, 2016, and 2017, respectively. Of these, 55.24% were women. In addition, 8.90%, 9.46%, and 8.37% of all patients in 2015, 2016, and 2017, who had applied for medical insurance, had CC, respectively, with a three-year average probability of 8.88%. The median number of outpatient visits within a calendar year was 27 per year due to any reason during the period of 2015–2017. The median medical cost of each patient per year increased from 935.30 USD to 1191.47 USD from 2015 to 2017.

**Conclusion:**

CC is common among medical insurance users, with a substantial utilization of medical resources, highlighting the huge burden of CC in China.

**Supplementary Information:**

The online version contains supplementary material available at 10.1186/s12890-024-03017-6.

## Background

Chronic cough (CC), defined as a cough lasting for more than eight weeks in adults [1], is a common symptom in the general population worldwide [2]. A meta-analysis has indicated a prevalence rate of 9.6% (95% confidence interval, CI: 7.6–11.7%) for CC in adults globally [3]. A higher prevalence of CC was reported in Oceania (18.1%, 95% CI: 9.8–27.2%), Europe (12.7%, 95% CI: 10.4–15.2%), and America (11.0%, 95% CI: 7.8–14.4%) than that in Asia (4.4%, 95% CI: 1.8–7.4%) [3]. A recent meta-analysis with 141,114 adults included estimated the prevalence of CC in China was at 6.22% (95% CI: 5.03%–7.41%) [4]. However, the studies included in this meta-analysis had employed inconsistent diagnostic criteria for CC [4]. In addition, the reported prevalence of CC in various regions of China is 2.0–28.3% according to several epidemiological studies [5–8]. The information on patients with CC was mainly achieved via survey, patient self-report, and physician diagnosis.

Previously regarded as a concomitant symptom of various respiratory diseases [9], it is now claimed that the presence of CC could denote a clinical syndrome with distinct and intrinsic pathophysiology [10]. CC poses a significant health issue owing to its substantial impact on quality of life [11]. Patients with CC may also suffer from a high economic burden owing to reduced productivity and increased medical cost. A cross-sectional study in Finland demonstrated that CC decreased the quality of life and increased the economic burden because of increased number of visits to the physician and sick leave days [12]. A study based on administrative data from the United States showed that health-care resource utilization (HCRU) and the use of prescription medications of patients with CC were higher than those by matched patients without CC [13]. However, the economic burden of CC in China has not yet been studied.

Insurance claims data could provide useful information about health-care beneficiaries with CC. However, using such data to identify patients with CC has its challenges because, at present, CC is not a standard diagnosis. The CC patients were identified based on repetitive cough-related events. To avoid the possibility of underestimating the true prevalence of CC, we mainly investigated the number of times urban patients with CC used medical insurance and the corresponding economic burden they had to face.

## Methods

### Study design

This was a retrospective observational cohort study conducted to estimate the prevalence and the corresponding economic burden on patients with CC in urban China, using data extracted from China Health Insurance Research Association (CHIRA) in China. CHIRA is an administrative database initiated in 2007 and managed by the China Health Insurance Research Association [14, 15]. Information on every hospital visit and medical service claim was recorded in the database. The claims data were generated from the nationwide urban employee and resident basic medical insurance scheme, which covers 95% of the country’s population. However, rural residents, unemployed people, and people who do not receive basic medical insurance plans were not included in this database. Consequently, we could not include these people in our study.

**Fig. 1:**
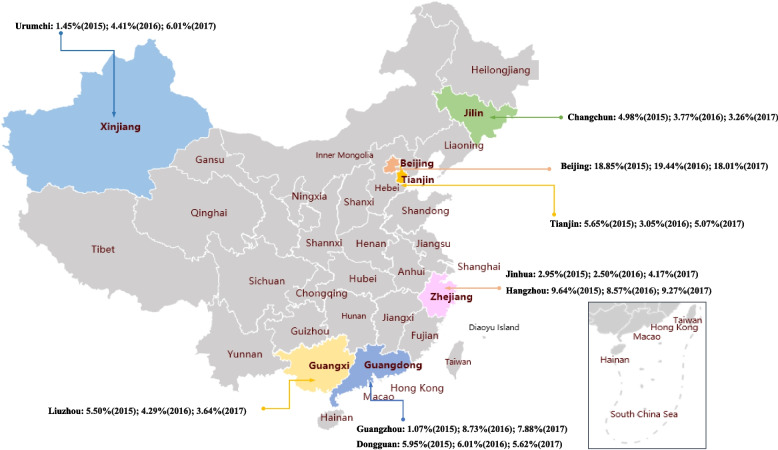
Geographic location of nine cities covered by the medical insurance database

This medical insurance database covers claims data from nine cities of the country (Beijing, Changchun, Dongguan, Guangzhou, Hangzhou, Jinhua, Liuzhou, Tianjin, and Urumchi [Wulumuqi]), located in the Northern (Beijing, Changchun, Tianjin, and Urumchi) and Southern (Dongguan, Guangzhou, Hangzhou, Jinhua, and Liuzhou) regions of China (Fig. 1). The nine cities were selected based on the following criteria: cities with diagnostic completeness lower than the 75th percentile (diagnostic completeness below 81.4%) were excluded and cities with median of annual number of visits per capita less than or equal to 2 visits were excluded. Administrative records based on a calendar year were retrieved from the hospital information systems of respective hospitals/health-care institutions and were used to set up the annual claims database.

The cohort identification period was set from January 1st to December 31st of 2015, 2016, and 2017, respectively. The individual-level observational period began from the first observed CC-related record till the end of each calendar year. All cough-related records were identified during the observational period (We have defined the cough-related and CC-related records in the section “Study population”). The study was conducted in accordance with the Helsinki Declaration. The study protocol was reviewed and approved by the Ethical Committee of the First Affiliated Hospital of Guangzhou Medical University. Only secondary analysis of the already anonymized data was conducted. Therefore, the need for written individual informed consent was waived by the ethical committee.

### Study population

As chronic cough has not been categorized as a claimable diagnosis in the medical system of all hospitals in China due to the inconsistencies in ICD code system/versions applied across the country, we adapted the algorithm for defining CC cases proposed by Holden et al. [16] which is developed to define CC by identifying probable and possible chronic cough (CC) from repeated cough records consistent with the established definition of CC in retrospective observational study using UK primary care database (Clinical Practice Research Datalink). Patients with ≥ 1 cough event in the study period were selected. Adults (aged ≥ 18 years) were classified as having probable CC if they had an explicit CC diagnosis; as having possible CC if they had ≥ 3 cough events recorded over 8–26 weeks. We made some adjustments to published methods owing to differences in the nature of the data source and the clinical setting/management practice and case-defining algorithm, as detailed below.

**Fig. 2:**
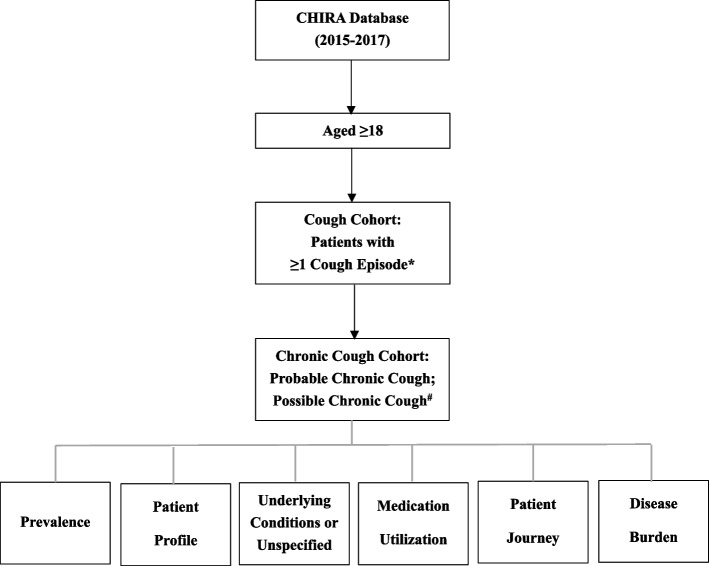
Study flow diagram

Firstly, this study identified all patients with any cough-related records (Supplementary Table 1) (hereinafter referred to as “cough visits”) and who were ≥ 18 years of age at the onset date of the first observed cough visit within the calendar years of 2015, 2016, and 2017, respectively. We excluded patients with missing information (Fig. 2). Secondly, patients with CC-related records (Supplementary Table 2) or health-care utilization patterns were identified.

The eligible patients were further defined as with “probable” or “possible” CC.

Patients with probable CC were defined as those with ≥ 1 diagnosis/condition/symptom record of chronic cough, persistent cough, cough hypersensitivity syndrome, refractory cough, prolonged cough, unknown chronic cough, psychogenic cough, or habit cough. It was defined based on the presence of “chronic cough” specific diagnosis record, therefore it is more clinically diagnosed and recorded chronic cough episode.

Patients with possible CC, on the other hand, without a “chronic cough” specific diagnosis, were defined as those with ≥ 3 cough visits within a period of 120 days during the study period.

Patients with CC were defined as the combination of probable CC and possible CC.

Patients with any previous cough visit 21 days before the start of the study and those in which the interval between the first and last cough visits was longer than eight weeks (56 days) were excluded. A minimum of 120-day observational period is required for defining CC and for outcome capturing. Therefore, patients who had their first cough visit after September 3rd (resulting in an observational period of less than 120 days before the calendar year ends) were also excluded from CC analyses.

We used the following criteria to define a cough visit: 1) a record of hospitalization or an outpatient clinic visit with a diagnosis/condition/symptom description of cough or cough-related condition, 2) a prescription record of a drug specifically indicated for cough, or 3) a prescription record of Chinese medicinal products under the category of cough suppressant under insurance formulary during the observational period (a detailed list is provided in Supplementary Table 3).

Observation period starts from the reference date of first eligible cough diagnosis (as Day 1), and ends at: 1) the reference date of the last eligible cough diagnosis within the calendar year, or 2) Day 120, whichever is later.

A list of comorbidities of interest was selected. These medical conditions, as reported in previous studies, were commonly associated with presence of chronic cough [13, 17–20]. These medical conditions include but not limited to cough-variant asthma (CVA), upper airway cough syndrome (UACS), eosinophilic bronchitis (EB), atopic cough (AC), gastro-esophageal reflux disease (GERD), and chronic obstructive pulmonary disease (COPD). Prescription of angiotensin-converting enzyme inhibitors (ACEI) during observation period were also assessed. Similar as cough diagnosis/treatment identification, comorbidities and ACEI prescription status were defined by presence in diagnosis record or prescription record. List of keywords and local languages used to identify comorbidities and ACEI drugs are listed in Supplementary Table 4 and 5. Due to the potential missing and delay in diagnosis making, diagnosis or prescription recorded 90 days before observation period start to 90 days after observation period end were considered as relevant comorbidities records.

### Outcome measures and statistical analysis

The primary outcome of the present study is the prevalence of CC, which was determined by dividing the number of patients with CC identified in each calendar year subset by the total number of individuals in that subset during the corresponding time periods for 2015, 2016, and 2017, respectively. The secondary outcome measures were the economic burden resulting from CC, including medical costs and HCRU outcomes, and HCRU outcomes comprise of number of outpatient visits due to any reason (not only due to cough), number of hospitalizations, and length of hospitalization(s). Economic burden was defined as all direct medical cost recorded in the claim database (not limited to CC related cost) incur during the observation period. The medication cost was defined as all medication cost recorded in the claim database. To sum up the length of hospitalization(s), all hospitalizations from the date of admission falling within the course of chronic cough were counted. Total and categorized direct medical expense included expenses incurred by medications, examinations, procedures, hospitalizations (if any), and use of medical devices in cough visits with the date of reference within the disease course of CC in an individual patient.

Statistical analysis was performed using SAS (SAS 9.4). No statistical hypothesis testing was performed. Continuous variables were described as the mean with standard deviation (SD) or median with the first and third quartiles. Categorical variables were described in frequency and proportion in this study.

## Results

### The proportion of subjects with CC among the total population in the CHIRA database

The records of 499,944, 587,241, and 674,204 patients were acquired from the database in 2015, 2016, and 2017, respectively (Table 1). Among these, A total of 44,472 patients in 2015, 55,565 in 2016, and 56,439 in 2017 were identified to have CC. The prevalence of CC was 8.90% in 2015, 9.46% in 2016, and 8.37% in 2017 among the study population. The average prevalence weighted by the sampled population in the three years was 8.88%.

**Table 1:** Number of patients with chronic cough and the prevalence of chronic cough

	**Cohort 2015**	**Cohort 2016**	**Cohort 2017**
Patients with at least one record in the database	499,944	587,241	674,204
Probable chronic cough patients (%)	31,213 (70.19)	36,920 (66.44)	37,781 (66.94)
Possible chronic cough patients (%)	13,259 (29.81)	18,645 (33.56)	18,658 (33.06)
Chronic cough patients^a^	44,472	55,565	56,439
Prevalence (as a proportion of patients in the database)^b^	8.90%	9.46%	8.37%

^a^The number of chronic cough patients was defined as the sum of probable chronic cough patients and possible chronic cough patients

^b^Prevalence was defined as the number of chronic cough patients as a proportion of the patients in the claims database in corresponding years

### Patient profiles

The patient characteristics, as summarized in Table 2, show that the mean age of the included patients in 2015, 2016, and 2017 was around 53 years. The proportion of women was slightly higher than men in all three years. The majority of patients belonged to the northern region of the country, and most patients with CC had UEBMI. The percentage of patients with URBMI was relatively small. These characteristics remained relatively consistent over the three years, with slight variations in certain parameters such as the proportion of patients from the northern region and the type of medical insurance. The comorbidities of patients with CC are summarized in Table 2. In general, 72.43%, 55.30%, and 69.74% of patients in 2015, 2016, and 2017, respectively, had at least one comorbidity before they were diagnosed with CC. The top three comorbidities were chronic pharyngitis, chronic bronchitis, and rhinitis and sinusitis. These three comorbidities were more prevalent among patients with probable CC compared to those in patients with possible CC. The proportions of patients with chronic pharyngitis were 40.72%, 27.95%, and 36.75% in 2015, 2016, and 2017, respectively; those with chronic bronchitis were 28.87%, 25.69%, and 29.24% in 2015, 2016, and 2017, respectively; and those with rhinitis and sinusitis were 8.46%, 6.61%, and 8.32% in 2015, 2016, and 2017, respectively. Notably, this proportion remained consistent for all three years.

**Table 2:** Profiles of patients with chronic cough

Variable	Cohort 2015 (*N* = 44,472)	Cohort 2016 (*N* = 55,565)	Cohort 2017 (*N* = 56,439)
**Age**			
Mean (SD)	53.2 (16.3)	53.2 (16.4)	53.8 (16.4)
Median (IQR)	54 (40–65)	54 (39–65)	54 (40–65)
**Sex**			
Men, *n* (%)	19,906 (44.76)	24,801 (44.63)	25,362 (44.94)
Women, *n* (%)	24,566 (55.24)	30,764 (55.37)	31,077 (55.06)
**Comorbidities**			
Patients had at least one of the following comorbidities in the baseline, *n* (%)	32,209 (72.43)	30,726 (55.30)	39,359 (69.74)
CVA, *n* (%)	162 (0.36)	282 (0.51)	365 (0.65)
UACS, *n* (%)	4 (0.01)	8 (0.01)	11 (0.02)
EB, *n* (%)	1 (0.00)	0 (0.00)	1 (0.00)
AC, *n* (%)	163 (0.37)	221 (0.40)	242 (0.43)
GERD/GERC, *n* (%)	2413 (5.43)	2638 (4.75)	3182 (5.64)
COPD, *n* (%)	1501 (3.38)	1445 (2.60)	1749 (3.10)
Chronic pharyngitis, *n* (%)	18,111 (40.72)	15,533 (27.95)	20,743 (36.75)
Bronchiectasis, *n* (%)	487 (1.10)	413 (0.74)	633 (1.12)
Asthma, *n* (%)	2049 (4.61)	2067 (3.72)	2743 (4.86)
Lung cancer, *n* (%)	131 (0.29)	114 (0.21)	160 (0.28)
Chronic bronchitis, *n* (%)	12,837 (28.87)	14,277 (25.69)	16,502 (29.24)
Respiratory tuberculosis, *n* (%)	114 (0.26)	73 (0.13)	115 (0.20)
Rhinitis and sinusitis, *n* (%)	3761 (8.46)	3675 (6.61)	4697 (8.32)
Whooping cough, *n* (%)	1 (0.00)	0 (0.00)	0 (0.00)
CCHS, *n* (%)	0 (0.00)	0 (0.00)	0 (0.00)
Patients had none of the above-listed comorbidities in the baseline, *n* (%)	12,263 (27.57)	24,839 (44.70)	17,080 (30.26)
**Residential region**			
North, *n* (%)	31,223 (70.21)	37,793 (68.02)	34,797 (61.65)
South, *n* (%)	13,249 (29.79)	17,772 (31.98)	21,642 (38.35)
**Insurance scheme type**			
UEBMI, *n* (%)	42,888 (96.44)	53,513 (96.31)	53,534 (94.85)
URBMI, *n* (%)	1584 (3.56)	2052 (3.69)	2905 (5.15)

*IQR* Interquartile range, *UEBMI* Urban employee basic medical insurance, *URBMI* Urban resident basic medical insurance, *CVA* Cough-variant asthma, *UACS* Upper airway cough syndrome, *EB* Eosinophilic bronchitis, *AC* Atopic cough, *GERD/GERC* Gastro-esophageal reflux disease/ gastro-esophageal reflux-related cough, *COPD* Chronic obstructive pulmonary disease, *CCHS* Congenital central hypoventilation syndrome

### Health-care resource utilization

Utilization of clinical resources by patients with CC is summarized in Table 3. In 2015 and 2016, the patients visited the outpatient departments 27 times due to any reason to seek treatment during their CC episodes (irrespective of whether it was cough-related or not). The median number of such visits was 27 in 2017. However, we would like to emphasize that every patient visit to the hospital was not due to a CC attack. Some of these patients were hospitalized during their episodes of CC.

**Table 3:** Utilization of health-care resources by patients with chronic cough

Variable	Cohort 2015	Cohort 2016	Cohort 2017
**Number of outpatient visits per year**			
* N*	44,472	55,565	56,439
Median (IQR)	27.43 (15.00–45.00)	27.00 (15.00–42.00)	27.00 (15.00–42.00)
**Number of inpatient visits per year**			
Number and proportion of patients with at least one hospitalization, *n* (%)	3956(8.9)	5314(9.6)	5696(10.1)
Median (IQR)	3.00 (3.00–3.83)	3.00 (3.00–4.65)	3.00 (3.00–5.03)
**Length of hospital stay (day) per year**			
* N*	3956	3758	5696
Median (IQR)	30.00 (18.00–48.00)	27.50 (15.00–48.00)	27.00 (15.00–45.77)
**Length of hospital stay (day) per hospitalization**			
* N*	3956	3758	5696
Median (IQR)	9.00 (6.00–13.00)	9.00 (6.00–13.00)	9.00 (5.00–12.5)

*SD* Standard deviation, *Q1* 25th percentile, *Q3* 75th percentile

In 2015 and 2016, the median number of hospitalizations was three, the total length of hospital stay was 30 and 27.5 days, respectively, and the length of hospital stay per hospitalization was 9 days. In 2017, the median number of hospitalizations was also 3, length of stay was 27 days per year, and length of hospital stay per hospitalization was 9 days per admission.

### Medical cost

Medical costs borne by all patients with CC are summarized in Table 4. In 2015 and 2016, the median total medical cost per year was 935.30 USD and 1157.49 USD, respectively, and the cost per admission was 1181.78 USD and 1404.98 USD, respectively. In 2015 and 2016, the median medication cost per year was 1057.68 USD and 855.78 USD, and the median non-medication cost per year was 0 and 164.00 USD, respectively. In 2015 and 2016, the median medical cost per outpatient per year was 338.63 USD and 468.37 USD, respectively. In 2017, the median total medical cost was 1191.47 USD, the median medical cost per outpatient was 492.14 USD, and the cost per admission was 1360.90 USD. The median medication and non-medication costs in 2017 were 813.19 USD and 230.51 USD, respectively.

**Table 4:** Medical costs for treating patients with chronic cough

	**Cohort 2015**	**Cohort 2016**	**Cohort 2017**
**Total medical cost per year**^**a**^** (USD)**			
* N*	44,472	55,565	56,439
Mean (SD)	2045.65 (4328.43)	2564.35 (5622.42)	2650.53 (6363.02)
Median (IQR)	935.30 (228.04–2382.87)	1157.49 (436.62–2886.14)	1191.47 (426.09–2994.08)
**Medication cost per year**^**a**^** (USD)**			
* N*	44,472	55,565	56,439
Mean (SD)	1898.41 (3086.87)	1654.42 (2804.87)	1582.86 (2870.68)
Median (IQR)	1057.68 (380.17–2463.70)	855.78 (306.61–2106.49)	813.19 (280.89–2037.62)
**Non-medication cost per year**^**a**^** (USD)**			
* N*	44,472	55,565	56,439
Mean (SD)	420.71 (2173.69)	910.72 (3483.09)	1067.40 (4183.98)
Median (IQR)	0 (0–71.11)	164.00 (29.11–598.67)	230.51 (55.13–758.55)
**Medical cost for outpatient visit per year**^**a**^** (USD)**			
* N*	44,472	55,565	56,439
Mean (SD)	424.44 (435.68)	589.53 (509.03)	604.70 (537.12)
Median (IQR)	338.63 (145.00–600.02)	468.37 (254.44–783.11)	492.14 (263.30–808.54)
**Medical cost per hospitalization**^**b**^** (USD)**			
* N*	3956	5314	5696
Mean (SD)	1807.98 (2180.19)	2136.78 (2597.12)	2100.19 (3003.83)
Median (IQR)	1181.78 (640.34–2125.88)	1404.98 (830.74–2387.16)	1360.90 (765.40–2263.36)

CNY to USD Exchange Rate: 2015:6.2284; 2016:6.6401; 2017:6.7547

The economic burden was defined as all direct medical costs recorded in the claim database, incurred during the disease course of chronic cough (not limited to costs related to chronic cough)

The medication cost was defined as all medication costs recorded in the claim database

^a^The costs were calculated among all enrolled patients

^b^The costs were calculated among enrolled patients with at least one hospitalization

### Medication use

The top three medications remained the same for all three years. The three most commonly used medicines for CC were ambroxol (2015: 17.19%; 2016: 17.65%; 2017: 16.29%), methoxyphenamine (2015: 7.57%; 2016: 10.18%; 2017: 10.49%), and eucalyptol (2015: 6.43%; 2016: 7.07%; 2017: 7.64%), whereas the most common type of prescriptions were antibacterial drugs (2015: 74.05%; 2016: 70.87%; 2017: 70.85%), followed by cough and cold drugs (2015: 46.73%; 2016: 60.25%; 2017: 60.06%), and other hematologic drugs (2015: 34.07%; 2016: 33.07%; 2017: 32.23%). The proportions of patients using angiotensin-converting enzyme inhibitors (ACEI) were 9.97%, 8.67%, and 2.69% in 2015, 2016, and 2017, respectively. This proportion remained similar for all patients with CC, probable CC, and possible CC (Table 5).

**Table 5:** Medications prescribed to patients with chronic cough during the disease course

	**Cohort 2015**	**Cohort 2016**	**Cohort 2017**
**ACEI use**			
Yes, *n* (%)	4436 (9.97)	4816 (8.67)	1516 (2.69)
No, *n* (%)	40,036 (90.03)	50,749 (91.33)	54,923 (97.31)
**Cough medications**			
Benproperine, *n* (%)	1 (0.00)	9 (0.02)	13 (0.02)
Benzonatate, *n* (%)	0 (0.00)	0 (0.00)	0 (0.00)
Codeine, *n* (%)	976 (2.19)	1027 (1.85)	836 (1.48)
Dextromethorphan, *n* (%)	1679 (3.78)	2155 (3.88)	2191 (3.88)
Levodropropizine, *n* (%)	0 (0.00)	0 (0.00)	2 (0.00)
Methoxyphenamine compound, *n* (%)	3367 (7.57)	5657 (10.18)	5920 (10.49)
Moguisteine, *n* (%)	0 (0.00)	0 (0.00)	0 (0.00)
Noscapine, *n* (%)	0 (0.00)	0 (0.00)	9 (0.02)
Promethazine, *n* (%)	0 (0.00)	0 (0.00)	0 (0.00)
Pentoxyverine, *n* (%)	1028 (2.31)	919 (1.65)	622 (1.10)
Glycyrrhiza preparation, *n* (%)	567 (1.27)	785 (1.41)	979 (1.73)
Pseudoephedrine, *n* (%)	636 (1.43)	756 (1.36)	697 (1.23)
Baclofen, *n* (%)	29 (0.07)	36 (0.06)	38 (0.07)
Gabapentin, *n* (%)	79 (0.18)	124 (0.22)	135 (0.24)
Ambroxol, *n* (%)	7644 (17.19)	9810 (17.65)	9194 (16.29)
Cysteine, *n* (%)	1768 (3.98)	2350 (4.23)	2585 (4.58)
Bromhexine, *n* (%)	370 (0.83)	696 (1.25)	1004 (1.78)
Eucalyptol, *n* (%)	2859 (6.43)	3929 (7.07)	4311 (7.64)
Myrtol Standardized, *n* (%)	1153 (2.59)	778 (1.40)	635 (1.13)
Fudosteine, *n* (%)	13 (0.03)	13 (0.02)	45 (0.08)
Carbocisteine, *n* (%)	2460 (5.53)	3387 (6.10)	3144 (5.57)
Acetylcysteine, *n* (%)	1767 (3.97)	2340 (4.21)	2576 (4.56)
**Therapeutic class (Top 20)**			
Traditional Chinese Medicine, *n* (%)	43,360 (97.50)	53,637 (96.53)	53,952 (95.59)
Antibacterial drugs, *n* (%)	32,930 (74.05)	39,378 (70.87)	39,985 (70.85)
Cough and cold drugs, *n* (%)	20,780 (46.73)	33,477 (60.25)	33,895 (60.06)
Other hematologic drugs, *n* (%)	15,151 (34.07)	18,375 (33.07)	18,193 (32.23)
Calcium channel blockers, *n* (%)	14,664 (32.97)	17,441 (31.39)	17,713 (31.38)
Anti-inflammatory and antirheumatic drugs, *n* (%)	12,120 (27.25)	15,211 (27.38)	15,109 (26.77)
Drugs for obstructive airway diseases, *n* (%)	9335 (20.99)	13,280 (23.90)	14,499 (25.69)
Drugs for acid related disorders, *n* (%)	12,531 (28.18)	14,972 (26.95)	15,754 (27.91)
Antihistamines for systemic use, *n* (%)	10,867 (24.44)	13,911 (25.04)	14,648 (25.95)
Antithrombotic agents, *n* (%)	4676 (10.51)	15,227 (27.40)	15,952 (28.26)
Agents acting on the renin-angiotensin system, *n* (%)	12,579 (28.29)	15,279 (27.50)	15,671 (27.77)
Ophthalmological drugs, *n* (%)	10,583 (23.80)	11,213 (20.18)	13,391 (23.73)
Lipid modifying agents, *n* (%)	11,355 (25.53)	13,918 (25.05)	15,244 (27.01)
Vitamins, *n* (%)	15,958 (35.88)	12,303 (22.14)	11,868 (21.03)
Mineral supplements, *n* (%)	4369 (9.82)	11,846 (21.32)	11,285 (20.00)
Cardiac therapy, *n* (%)	10,222 (22.99)	9991 (17.98)	10,084 (17.87)
Analgesic drugs (N02), *n* (%)	23,932 (53.81)	8755 (15.76)	8148 (14.44)
Antidiarrheals, intestinal anti-inflammatory/anti-infective agents, *n* (%)	6673 (15.00)	6467 (11.64)	8281 (14.67)
Drugs used in diabetes, *n* (%)	8612 (19.36)	9891 (17.80)	10,323 (18.29)
Antianemic drugs, *n* (%)	7070 (15.90)	8211 (14.78)	8448 (14.97)
**Therapeutic class R**			
Nasal preparations, *n* (%)	3486 (7.84)	3338 (6.01)	3508 (6.22)
Drugs for obstructive airway diseases, *n* (%)	9335 (20.99)	13,280 (23.90)	14,499 (25.69)
Cough and cold drugs, *n* (%)	20,780 (46.73)	33,477 (60.25)	33,895 (60.06)
Antihistamines for systemic use, *n* (%)	10,867 (24.44)	13,911 (25.04)	14,648 (25.95)
Other respiratory system products, *n* (%)	187 (0.42)	537 (0.97)	559 (0.99)

*ACEI* Angiotensin-converting enzyme inhibitor

## Discussion

Clinical diagnosis and the management of CC are usually challenging owing to its cross-specialty, prolonged course, and low awareness among population [21]. Therefore, CC remains underdiagnosed in China, leading to an underestimation of its potential clinical and economic burden in the country. This may explain why the epidemiology of CC and related clinical outcomes has not been well studied in China.

To the best of our knowledge, this retrospective study is the first to evaluate the prevalence and economic burden of CC using an insurance claims database in nine cities in China. Between 2015 and 2017, 8.37–9.46% of those who received medical insurance had CC. This study uses an insurance claims database to estimate the health-care utilization and economic burden caused by CC in China. This approach helps understand the perspectives of both a patient and the health-care system because of the long-term disease course and continuous hospital visits [22]. However, not all subjects with CC will seek medical advice, purchase medication, or receive medical insurance. Sun et al. (2021) reported that 19.3% of participants (1129/5855) had a cough in the past month, while only 40% (452/1129) had sought medical treatment [17]. Fujimura et al. (2012) reported that more than 60% of the surveyed individuals did not receive any care and 44.0% had no plans to visit a medical facility [18]. The use of the current method will lead to an underestimation of the prevalence of CC. Therefore, the incidence rate of CC proposed in this study does not represent the overall population prevalence. This issue requires special emphasis.

Regarding patient profiles, women and the northern region of China tend to have a higher prevalence of CC. As reported by a survey conducted in multiple countries (including Sweden, South Korea, China, UK, US, and Netherland) in 2014, CC was more prevalent in women (66.7%) [2]. Another study indicated that long-term exposure to dust could be responsible for the higher incidence of CC in women than that in men in China [5]. This higher prevalence of CC in women could also be partly due to the lower tolerance to cough triggers and increased sensitivity in the central processing of cough sensations in women [2]. In our study, the mean age of patients with CC was greater than that of patients in respiratory specialist clinics, probably because we included more patients from the community.

A recent meta-analysis reported a prevalence of 4.38% for CC in Southern China, while it was 8.70% in Northern China [4]. Similarly, we observed a higher prevalence of CC in Northern China than that in Southern China. This regional difference could be explained by the higher urbanization and greater severity of environmental pollution in Northern China [19].

CC greatly increases the disease burden of a country, and hence, it is a significant issue from both clinical and economic perspectives. As reported, CC negatively impacts the quality of life in China, Japan, Korea, the U.S., and Europe [20, 23–26]. Previous studies also showed an increase in the economic burden owing to CC. A US-based study using the KPSC Research Data Warehouse reported that patients with CC had significantly more HCRU, underwent more laboratory tests, and had a greater dispension of medications than those without CC [13]. An internet survey on the general population across Europe indicated that more than 70% of patients with CC had three or more physician visits [23]. According to the National Health and Wellness Survey in Japan, patients with CC reported 1.4 times more visits to health-care providers and six times more visits to emergency rooms in the past six months, compared with those by non-cough patients [24]. Despite the generally lower unit cost and shorter waiting time for medical appointment in China as compared with more developed countries and regions, the country still faced a similar increase in the utilization of health-care resources. Each patient with CC visited a hospital or clinic (for any reason) 27 times per year and utilized the outpatient service costing 339–492 USD per visit. In addition, patients with CC faced a huge economic burden. Compared with the annual total medical cost (263 USD) incurred by the urban population of China [27], the median total medical cost of patients with CC in 2017 was almost five times (2,650 USD) higher in our study. These results were similar to those reported by previous studies from other countries.

However, it should be noted that not every hospital visit is due to CC. The visits could be due to complications arising from hypertension, diabetes, coronary heart disease, and other comorbidities. The patients may also visit the hospital regularly for medication. This may increase the average frequency of visits for the overall patient population.

Data published in *The Lancet* 2019 show that per capita health-care expenditure in China had increased year on year from 2015 to 2017 [28] (401.5 USD, 436.1 USD, and 464.9 USD, respectively). Some studies have also investigated the cost of treating chronic diseases in China. For example, in 2016, the average annual outpatient cost for patients with migraine was RMB 322 (48.5 USD), of which medicines alone accounted for RMB 249 (37.5 USD) [29]. In 2015, the average annual mean cost for the treatment and management of diabetes was RMB 46,324 (6,976.5 USD) [30]; in 2015, the average annual direct medical cost for chronic kidney disease (CKD) along with anemia was RMB 29,459 (4,436.6 USD) [31]; and again in 2015, the average outpatient cost for patients with osteoporosis in China was RMB 493.32 (74.3 USD). In contrast, the average annual inpatient cost for patients with osteoporosis was RMB 18,254.31 (2,749.1 USD) [32] under the exchange rate of 6.64 (US Dollar/Chinese Yuan Renminbi) as captured on year/month/day. CC, although not a stand-alone disease, also increases the health-care costs for patients. However, no data on this aspect are yet available, leading to under-reporting of its potentially significant clinical and economic burden.

A major strength of the present study is that it updated national data on the prevalence of CC in China. Because the available data were generated from surveys and meta-analyses, the use of claims data and a large sample size could make the study more representative and useful for prevalence estimation. We developed a patient-identification algorithm for a comprehensive review of CC. This is the first study to examine the HCRU and medical cost of CC using a national claims database in China.

The study has several limitations. Firstly, owing to the nature of the annual-based sampling method of the claims database, the observational period had to be segmented at the end of a calendar year. Follow-up from one calendar year to the next one could not be performed and as a result, seasonality pattern of disease was not assessed in this study. Secondly, episodes of CC during the last 120 days of each calendar year were excluded, which may lead to an underestimation of the prevalence of CC owing to its seasonal pattern. In addition, patients with CC may also seek medical attention because of other diseases/conditions. Thirdly, tobacco use or OTC drugs use had an effect on the epidemiology of chronic cough yet due to the nature of the data source used in this study, these effects were not able to be measured. Fourthly, we intended to include as much as possible quality research-ready data to maximize representativeness at the time we performed data collection (2019-early 2020). The nine cities were selected based on data completeness rate among all. We acknowledged that due to data availability, representativeness of the results might be limited. Lastly, the results should be more cautiously generalized, because the studied population comprised urban employees and residents in the claims database, instead of the general population. Therefore, the incidence rate obtained in this study will be lower than that in the total population of China.

## Conclusions

In summary, this is the first study to evaluate the prevalence of CC and disease burden caused by CC in urban China using a nationwide claims database. These results highlight that the burden of CC in China was significant, and that a noticeable amount of health-care resources was utilized. Additionally, patient behavior analysis based on medical insurance data was explored, which may assist in identifying potential risk groups and help individual diagnosis of CC in China. Because the sample included in this study comprises only the subjects in the national claim database, further research on the prevalence and disease burden of CC in the overall population of China is needed.

### Supplementary Information

**Supplementary Material 1.**

## Data Availability

The data that support the findings of this study are available from the corresponding author upon reasonable request.

## Abbreviations

CC: Chronic cough
SD: Standard deviation
