# Supplementary material for: Prevalence and disease burden of chronic cough in nine cities of China: an observational study
Source: BMC Pulm Med. 2024 Jul 4;24:322. doi: 10.1186/s12890-024-03017-6 (PMC11225231; doi:10.1186/s12890-024-03017-6)
Supplement: Supplementary file 1 — Supplementary Material 1. [file 12890_2024_3017_MOESM1_ESM.docx]

**Supplementary Table 1.** Cough related diagnosis/condition/symptom for cough episode identification

| **Cough episode: cough related diagnosis/condition/syndrome** |
| --- |
| Cough |
| Hemoptysis |
| Whooping cough |
| Cold |
| Flu |
| Acute rhinitis |
| Acute nasopharyngitis |
| Acute sinusitis |
| Bacterial sinusitis |
| Suppurative sinusitis |
| Acute pharyngitis |
| Acute tonsillitis |
| Acute tonsil abscess |
| Acute laryngitis |
| Acute bronchitis |
| Acute epiglottitis |
| Acute upper respiratory tract infection |
| Influenza |
| Pneumonia |
| Acute bronchitis |
| Acute bronchiolitis |
| Acute lower respiratory tract inflammation |
| Bacterial rhinitis |
| Acute obstructive laryngitis |

The diagnosis/condition/symptom were defined by keyword searching within diagnosis data field. ICD coding was not available in the claim database, and it was not being used in diagnosis identification.

**Supplementary Table 2.** Probable chronic cough related diagnosis/condition/symptom for cough episode identification

| **Chronic cough related diagnosis/condition *(******for Probable chronic cough)*** |
| --- |
| Atopic cough |
| Chronic cough |
| Bronchiectasis |
| Chronic bronchitis |
| Chronic pharyngitis |
| Chronic cough hypersensitivity syndrome |
| Cough-variant asthma |
| Eosinophilic bronchitis |
| Gastro-esophageal reflux cough |
| Prolonged cough |
| Postnasal drip syndrome |
| Psychogenic cough/habit cough |
| Refractory cough |
| Unknown/unspecified cough/chronic cough |
| Upper airway cough syndrome |

The diagnosis/condition/symptom were defined by keyword searching within diagnosis data field. ICD coding was not available in the claim database, and it was not being used in diagnosis identification.

**Supplementary Table 3.** Drugs and Chinese medicinal products indicated for cough episode identification

|  | **Drug name** |
| --- | --- |
| Cough related medication | Benproperine |
|  | Benzonatate |
|  | Codeine |
|  | Dextromethorphan |
|  | Levodropropizine |
|  | Methoxyphenamine compound |
|  | Moguisteine |
|  | Noscapine |
|  | Promethazine |
|  | Pentoxyverine |
|  | Glycyrrhiza preparation |
|  | Pseudoephedrine |
|  | Baclofen |
|  | Gabapentin |
|  | Ambroxol |
|  | Cysteine |
|  | Bromhexine |
|  | Eucalyptol |
|  | Myrtol Standardized |
|  | Fudosteine |
|  | Carbocisteine |
|  | Acetylcysteine |
| Chinese medicinal product under the category of cough suppressant* | 祛痰止咳颗粒 (QuTanZhiKeKeLi) |
|  | 蛇胆陈皮(散/胶囊/片) (SheDanChenPi (San/JiaoNang/Pian)) |
|  | 蛇胆川贝液 (SheDanChuanBeiYe) |
|  | 消咳喘(颗粒/胶囊/片) XiaoKeChuan (KeLi/JiaoNang/Pian) |
|  | 白百抗痨颗粒 (BaiBaiKangLaoKeLi) |
|  | 金荞麦(胶囊/片) (JinQiaoMai (JiaoNang/Pian)) |
|  | 克咳胶囊(片) (KeKeJiaoNang (Pian)) |
|  | 利肺片 (LiFeiPian) |
|  | 牛黄蛇胆川贝(散/胶囊/片/滴丸/液) (NiuHuangSheDanChuanBei(San/JiaoNang/Pian/DiWan/Ye)) |
|  | 清宣止咳颗粒 (QingXuanZhiKeKeLi) |
|  | 祛痰灵口服液 (QuTanLingKouFuYe) |
|  | 祛痰止咳胶囊 (QuTanZhiKeJiaoNang) |
|  | 蛇胆陈皮口服液 (SheDanChenPiKouFuYe) |
|  | 蛇胆川贝(散/胶囊) (SheDanChuanBei (San/JiaoNang)) |
|  | 蛇胆川贝枇杷膏(SheDanChuanBeiPiPaGao) |
|  | 苏黄止咳胶囊(SuHuangZhiKeJiaoNang) |
|  | 痰咳净(散/片)(TanKeJing (San/Pian)) |
|  | 消咳喘糖浆(XiaoKeChuanTangJiang) |
|  | 宣肺止嗽合剂(XuanFeiZhiSouHeJi) |
|  | 止咳(丸/片) (ZhiKe (Wan/Pian)) |
|  | 治咳川贝枇杷(露/滴丸) (ZhiKeChuanBeiPiPa (Lu/DiWan)) |
|  | 二陈丸(ErChenWan) |
|  | 橘红痰咳煎膏（颗粒/液）(JuHongTanKeJianGao (KeLi/Ye)) |
|  | 养阴清肺丸 (YangYinQingFeiWan) |
|  | 治咳川贝枇杷露（滴丸）(ZhiKeChuanBeiPiPaLu (DiWan)) |
|  | 蜜炼川贝枇杷膏(MiLianChuanBeiPiPaGao) |
|  | 枇杷叶膏(PiPaYeGao) |
|  | 养阴清肺膏（糖浆/颗粒/口服液）(YangYinQingFeiGao (TangJiang/KeLi/KouFuYe)) |

* Chinese medicinal products under the category of cough suppressant in the China national healthcare insurance formulary in effect during the study period (2015-2017) are included. For Chinese medicinal products, drug names are provided only in local language.

**Supplementary Table 4.** Drug Indicated for Cough and Chinese Traditional Medicinal Products under the Category of Cough Suppressant in Local Language

| Generic Name | Generic Name in Chinese |
| --- | --- |
| Benproperine | 苯丙哌林 |
| Benzonatate | 苯佐那酯 |
| Codeine | 可待因 |
| Dextromethorphan | 右美沙芬 |
| Levodropropizine | 左羟丙哌嗪 |
| Methoxyphenamine | 复方甲氧那明 |
| Moguisteine | 莫吉司坦 |
| Noscapine | 那可 |
| Promethazine | 二氧丙嗪 |
| Pentoxyverine | 喷托维林 |
| Glycyrrhiza preparation | 复方甘草制剂 |
| Pseudoephedrine | 美敏伪麻 |
|  |  |
| (Chinese traditional medicinal product under the category of cough suppressant) ^a^ | |
| / | 祛痰止咳颗粒 |
| / | 蛇胆陈皮（散/胶囊/片） |
| / | 蛇胆川贝液 |
| / | 消咳喘（颗粒/胶囊/片） |
| / | 白百抗痨颗粒 |
| / | 金荞麦（胶囊/片） |
| / | 克咳胶囊（片） |
| / | 利肺片 |
| / | 牛黄蛇胆川贝（散/胶囊/片/滴丸/液） |
| / | 清宣止咳颗粒 |
| / | 祛痰灵口服液 |
| / | 祛痰止咳胶囊 |
| / | 蛇胆陈皮口服液 |
| / | 蛇胆川贝（散/胶囊） |
| / | 蛇胆川贝枇杷膏 |
| / | 苏黄止咳胶囊 |
| / | 痰咳净（散/片） |
| / | 消咳喘糖浆 |
| / | 宣肺止嗽合剂 |
| / | 止咳（丸/片） |
| / | 治咳川贝枇杷（露/滴丸） |
| / | 杏贝止咳颗粒 |
| / | 强力枇杷（膏/蜜炼/胶囊/颗粒） |
| / | 标准桃金娘油肠溶胶囊 |
| / | 小儿咳喘颗粒 |
| / | 急支颗粒 |
| / | 急支糖浆 |
| / | 小儿宣肺止咳颗粒 |

a Chinese medicinal product under the category of cough suppressant in the China national healthcare insurance formulary in effect during the study period (2015-2017) are included. For Chinese medicinal products, drug names are provided only in local language.

**Supplementary Table 5.** Chronic Cough Related Underlying Conditions or Comorbidities and Searching Terms in Local Language

| Common Underlying Conditions | Diagnosis/Condition/Syndrome | Keywords to Search | Translation |
| --- | --- | --- | --- |
| Atopic cough | Atopic cough | 变应性咳嗽 | Atopic cough |
|  |  | AC | Atopic cough (abbreviation) |
| Chronic obstructive pulmonary disease | Chronic bronchitis | 慢性支气管炎 | Chronic bronchitis |
|  | Chronic obstructive pulmonary disease | 慢性阻塞性肺病 | Chronic obstructive pulmonary disease |
|  |  | 慢阻肺 | Chronic obstructive pulmonary disease (abbreviation) |
|  |  | COPD | Chronic obstructive pulmonary disease (abbreviation) |
|  | Emphysema | 肺气肿 | Emphysema |
| Cough-variant asthma | Cough-variant asthma | 咳嗽变异*哮喘 | Cough-variant asthma |
|  |  | CVA | Cough-variant asthma (abbreviation) |
| Eosinophilic bronchitis | Eosinophilic bronchitis | 酸细胞性支气管炎 | Eosinophilic bronchitis |
|  |  | EB | Eosinophilic bronchitis (abbreviation) |
| Gastro-esophageal reflux disease | Gastro-esophageal reflux disease | GERC | Gastro-esophageal reflux caused cough |
|  |  | *食管反流* | (abbreviation) |
|  |  | GERD | Gastro-csophageal reflux disease |
|  |  | 反流性食管炎 | Gastro-esophageal reflux disease (abbreviation) |
| Heart failure | Heart failure | *心功能衰竭 | Reflux esophagitis |
|  |  | 心脏衰竭 | Heart failure |
|  |  | 心衰 | Heart failure (abbreviation) |
| Irritable bowel syndrome | Irritable bowel syndrome | 肠易激* | Irritable bowel syndrome |
| Lung cancer | Malignant neoplasm of bronchus and lung | 肺鳞癌 | Lung squamous cell carcinoma |
|  |  | 肺腺癌 | Lung adenocarcinoma |
|  |  | 肺恶性肿癌 | Malignant neoplasm of bronchus and lung |
|  |  | *肺癌 | Malignant neoplasm of bronchus and lung (abbreviation) |
|  |  | SCLC | Small cell lung cancer (abbreviation) |
| Other specified conditions | Pertussis/Whooping cough | 百日咳 | Whooping cough |
|  | Respiratory tuberculosis | 支气管结核 | Bronchial tuberculosis |
|  |  | 肺结核 | Lung tuberculosis |
| Upper airway cough syndrome | Non-allergic rhinitis | 非过敏性鼻炎 | Non-allergic rhinitis |
|  | Sinusitis | 鼻窦炎 | Sinusitis |
|  | Upper airway cough syndrome | 上呼吸道咳嗽综合征 | Upper airway cough syndrome (abbreviation) |
|  |  | UACS | Post nasal drip syndrome |
|  |  | 鼻后滴流* | Post nasal drip syndrome (abbreviation) |
|  |  | PNDS* | Allergic rhinitis |
|  | Vasomotor and allergic rhinitis | 过敏性鼻炎 | Vasomotor rhinitis |
|  |  | 血管运动性鼻炎 | Vasoconstrictive rhinitis |
| Unspecified | Unknown chronic cough | 血管收缩性鼻炎 | Unknown chronic cough |
|  | Unspecified cough | 不明原因慢性咳嗽 | Unspecified cough |

“*”: wildcard term
